# Artificial Neural Network and Genetic Algorithm Hybrid Intelligence for Predicting Thai Stock Price Index Trend

**DOI:** 10.1155/2016/3045254

**Published:** 2016-11-15

**Authors:** Montri Inthachot, Veera Boonjing, Sarun Intakosum

**Affiliations:** ^1^Department of Computer Science, Faculty of Science, King Mongkut's Institute of Technology Ladkrabang, Bangkok 10520, Thailand; ^2^International College, King Mongkut's Institute of Technology Ladkrabang, Bangkok 10520, Thailand

## Abstract

This study investigated the use of Artificial Neural Network (ANN) and Genetic Algorithm (GA) for prediction of Thailand's SET50 index trend. ANN is a widely accepted machine learning method that uses past data to predict future trend, while GA is an algorithm that can find better subsets of input variables for importing into ANN, hence enabling more accurate prediction by its efficient feature selection. The imported data were chosen technical indicators highly regarded by stock analysts, each represented by 4 input variables that were based on past time spans of 4 different lengths: 3-, 5-, 10-, and 15-day spans before the day of prediction. This import undertaking generated a big set of diverse input variables with an exponentially higher number of possible subsets that GA culled down to a manageable number of more effective ones. SET50 index data of the past 6 years, from 2009 to 2014, were used to evaluate this hybrid intelligence prediction accuracy, and the hybrid's prediction results were found to be more accurate than those made by a method using only one input variable for one fixed length of past time span.

## 1. Introduction

Stock index, trend, and market predictions present a challenging task for researchers because movement of stock index is the result of many possible factors such as a company's growth and profit-making capacity, local economic, social, and political situations, and global economic situation. Good predictions are crucial for minimizing investment risk and maximizing return.

There are 2 kinds of stock analyses: fundamental and technical. The first kind is an analysis of the intrinsic value of a stock based on consideration of basic factors such as a company's growth and profit-making capacity, the growth of its industrial group, and the economic trend. The second kind, on the other hand, is a mathematical analysis based on past stock index records. The simplest analysis of this kind is to make prediction by observing stock movement trend in a graph. More sophisticated analyses employ complex statistical methods and machine learning algorithms.

Artificial Neural Network (ANN) is one of the popular machine learning algorithms that has been applied for time series forecasting and a widely accepted method for predictions of stock index, trend, and market [1, 2]. Kimoto et al. [3] were the first, in 1990, to apply a modular neural network machine learning algorithm to predict the movement of stock index of Tokyo Stock Exchange and the best times to buy and sell its stocks. Later on, ANN was developed and widely applied to stock analysis. For example, Wu and Lu [4] used ANN to predict S&P 500 stock index, compared its prediction results with those made by a Box-Jenkins model, and reported that ANN made more accurate predictions, while Zhang and Wu [5] used Improved Bacterial Chemotaxis Optimization (IBCO) with Backpropagation Neural Network (BPNN) to predict the same index. Birgul et al. [6] used ANN to predict ISE index. Bollen et al. [7] used data posted on Twitter to predict Dow Jones index. Guresen et al. [8] used 4 models—ANN Multilayer Perceptron (MLP), Dynamic Architecture for Artificial Neural Network (DAN2), GARCH-MLP, and GARCH-DAN2—to predict NASDAQ index and found that MLP was the most accurate. Wang et al. [9] combined Elman recurrent neural networks with stochastic time effective function to predict SSE, TWSE, KOSPI, and Nikkei225. Not only for predicting established stock markets, ANN was also used for predicting emerging ones. For example, Kara et al. [2] used ANN and SVM to predict the movement of Turkish ISE 100 index by importing several technical indicators and found that ANN's predictions were accurate. Patel et al. [10] proposed a preparation of trend deterministic data of technical indicators prior to import into models and found that it gave better prediction results than those given by a conventional import procedure when the indicators were imported into 4 models—ANN, SVM, Random Forest, and Naïve-Bayes classifier models—that were used to analyze CNX Nifty and S&P Bombay Stock Exchange markets. Manish and Thenmozhi [11] used ANN, SVM, logit, and Random Forest to predict the daily movement of direction of S&P CNX NIFTY Index and found that SVM outperformed the other models. In all of the works above, either ANN or SVM was the top performer. Most recently, Inthachot et al. [12] imported 10 technical indicators into ANN and SVM, used the models to predict the movement of Thailand's SET50 index, and found that ANN was more accurate than SVM, but both were still low in accuracy and needed to be further developed.

The Stock Exchange of Thailand (SET) is an emerging stock market in the TIP group (Thailand, Indonesia, and the Philippines) that has attracted the attention of Asian and global investors alike. When SET started to operate on April 30, 1975, only 16 public companies were registered; in 2015, the number exceeded 500. SET50 index is an index calculated from the stock prices of the top 50 companies registered in SET in terms of large market capitalization and high liquidity. Accurate prediction of SET50 index trend especially helps short-term investors to reduce risk and make profit from SET50 Futures and SET50 Index Options of the TFEX Futures markets.

As mentioned that the accuracy of SET50 index predictions based on technical indicators calculated from one past time span was still low, this study proposes importing technical indicators of which each is represented by 4 input variables based on 4 past time spans of different lengths—3-, 5-, 10-, and 15-day spans before the day of prediction—in order to generate more diverse subsets of input which is then culled down to a manageable number of effective ones by Genetic Algorithm (GA) and passed onto ANN to make prediction of SET50 index trend. Our contribution to application of GA to ANN was to use GA for finding a manageable number of effective subsets of input into ANN in order to improve the hybrid overall trend prediction accuracy.

The remainder of this paper is organized into the following sections: Section 2 is a literature review; Section 3 describes the methodology, research data, preprocessing of the data, prediction models, and measurement accuracy; Section 4 shows the experimental results and discussion; and Section 5 concludes the study.

## 2. Literature Review

This review focuses on several studies that have applied ANN to predict stock price and index in both established and emerging markets. Leung et al. [13] used various types of models based on multivariate classification method to predict stock index trend and reported that classification models (linear discriminant analysis, logit, probit, and probabilistic neural network) outperformed level estimation models (exponential smoothing, multivariate transfer function, vector autoregression with Kalman filter, and multilayered feedforward neural network) in terms of prediction accuracy of stock market movement direction and maximum return of investment trading. Chen et al. [14] used probabilistic neural network (PNN) to predict Taiwan Stock Exchange movement direction and applied the prediction to formulating trading strategies. They found that the prediction results obtained from PNN were more accurate than those obtained from GMM-Kalman filter and random walk. Altay and Satman [15] used ANN and linear regression to predict an emerging market movement direction and found that ANN gave more accurate predictions: 57.8%, 67.1%, and 78.3% for daily, weekly, and monthly data, respectively. Kara et al. [2] used ANN and SVM to predict Istanbul Stock Exchange (ISE) movement direction based on stock index data of 1997–2007 and employed 10 technical indicators as input variables—simple moving average, weighted moving average, momentum, stochastic K%, stochastic D%, RSI, moving average convergence divergence (MACD), Williams' R%, A/D oscillator, and CCI. The prediction accuracies of their ANN model were found to be 99.27% for the training data set and 76.74% for the test data set, while those of the SVM model were 100% for the training set but only 71.52% for the test data set. Chang et al. [16] used an evolving partially connected neural networks (EPCNNs) model and technical indicator input variables to predict the stock price movement of Taiwan Stock Exchange (TSE). The architecture of EPCNNs was different from that of ANN: connections between neurons were random; more than one hidden layer was accommodated; and weights were trained and adjusted with GA. They found that their proposed model gave more accurate predictions than those obtained from BPN, TSK fuzzy system, and multiple regression analysis. Patel et al. [10] proposed using deterministic input variables with ANN, SVM, Random Forest, and Naïve-Bayes models to predict Indian stock market index trend. They constructed a layer for converting 10 continuous input variables employed in a study by Kara et al. [2] into deterministic input variables before incorporating them into the models. The prediction results obtained from ANN, SVM, Random Forest, and Naïve-Bayes were 86.69%, 89.33%, 89.33%, and 90.19% accurate, respectively, which were higher than those obtained from models using continuous variables, the highest of which was 83.56% obtained from Random Forest model.

For the case of Thailand's stock market, Sutheebanjard and Premchaiswadi [17] used backpropagation neural network (BPNN) to forecast SET Index movement during July 2–December 30, 2004 (124 days), and obtained predictions with a mean square error (MSE) of 234.68 and a mean absolute percentage error (MAPE) of 1.96%. Inthachot et al. [12] applied ANN and SVM to predicting Thailand's SET50 index movement by employing the same 10 technical indicators that Kara et al. [2] used and the index data of 2009–2014 and found that year-by-year one-day prediction results by ANN were more accurate than those by SVM. ANN's average accuracy for this study was quite low, at 56.30%, when compared with the accuracy of the prediction results of ISE stock index movement which was most likely due to considerably wilder fluctuation of SET50 index values.

Readers who would like to have a comprehensive overview of recent stock market forecasting research studies should consult a review paper by Atsalakis and Valavanis [1].

## 3. Methodology

### 3.1. Data Preparation and Preprocessing

This work used a data set of daily SET50 index at closing time between January 5, 2009, and December 30, 2014 (1,464 days). During this period, the stock index moved up 795 times (54.30%) and down 669 times (45.70%), as shown in Table 1.

The data set was divided into 5 groups for 5-fold cross-validation runs, as shown in Table 2. A total of 5 runs were made in which each run used one group of data as a test data set and the other 4 groups as training data sets so that every group was used as a test data set exactly once.

From all of the widely accepted 11 technical indicators for stock price and index forecast [2, 10, 12, 18], each input variable was calculated by the equation of its corresponding indicator shown in Table 3. Four input variables were derived from each technical indicator in which each of the four variables was calculated based on one of these 4 past time span lengths: 3, 5, 10, and 15 days, making up a total of 11 × 4 = 44 input variables.

All input variables were normalized to [−1, 1] so that they all had the same weight. The only output variable could take a value of either 0 or 1—a value of 0 means that the predicted next-day SET50 index was lower than the prediction day index (down trend) while a value of 1 means that the predicted next-day index was higher than the prediction day index (up trend).

### 3.2. Prediction Models

#### 3.2.1. Artificial Neural Network (ANN)

ANN (introduced by McCulloch and Pitts [19]) is a machine learning model that mimics an aspect of human learning from past experience to predict a future outcome. ANN is widely adopted in research studies on stock price and index forecast [1, 2, 8, 16, 20]. It has already been used for predicting SET50 index trend [12] in a study and found to make more accurate predictions than support vector machine (SVM). However, its absolute accuracy was still not very good. This study attempted to develop it further to make it more accurate for predicting next-day SET50 index movement. Our ANN model was a three-layered feedforward model consisting of an input layer, a hidden layer, and an output layer. Past stock trading data were represented by 11 technical indicators. Each technical indicator was imported into ANN as 4 variables based on 4 different past time length spans, making up a total of 44 variables in the input layer. The number of nodes in the hidden layer was set to 100, following the optimum number used in a study by Inthachot et al. [12]. The transfer function between nodes in the input layer and the hidden layer and between nodes in the hidden layer and the output layer was tan sigmoid. The output layer had 1 neuron with log sigmoid transfer function. The computed output could take a value between 0 and 1 where a value equal to or less than 0.5 indicates a downward index movement and a value higher than 0.5 indicates an upward movement. A weight was assigned between each pair of connected nodes. Initially, all of the weights were randomly generated; then they were adjusted during the training period by a gradient descent with momentum method.

The model parameters that needed to be set were the number of hidden layer neurons (*n*), the learning rate (lr), the momentum constant (mc), and the number of iterations for learning (*ep*). They were set to *n* = 100, lr = 0.1, mc = 0.1, and *ep* = 8,000 following those that gave the best accuracy in the study by Inthachot et al. [12] mentioned above.

#### 3.2.2. A Hybrid Intelligence of ANN and Genetic Algorithm (GA)

ANN has several disadvantages such as long training time, unwanted convergence to local instead of global optimal solution, and large number of parameters; therefore, there have been attempts to remedy some of these disadvantages by combining ANN with another algorithm that can take care of a specific problem. An algorithm that has frequently been hybridized with ANN is GA. In 1990, Whitley et al. [21] began to use GA to optimize weighted connections and find a good architecture for neural network connections. In 2006, Kim [22] proposed a hybrid model of ANN with GA that performs instance selection to reduce dimensionality of data. In 2012, Karimi and Yousefi [23] used GA to find a set of weights for connections to each node in an ANN model and determine correlation of density in nanofluids. Sangwan et al. [24] proposed an integrated ANN and GA for predictive modeling and optimization of turning parameters to minimize surface roughness. Some other successful examples of ANN-GA hybrid applications are network intrusion detection [25] and cancer patient classification [26]. Inspired by these successes, this study attempted to use GA to solve a feature selection problem—to find effective subsets of input into ANN.

The rationale behind our idea of using a hybrid intelligence of ANN and GA was that it should be better to use, first, multiple input variables (4 in this study) for each technical indicator based on different past time spans (3, 5, 10, and 15 days) and, second, a small number of effective subsets of input variables that would be imported. Since the number of subsets of 44 variables is astronomical 2^44^, it would take too much computation time to process them. GA took care of that. GA is an algorithm that is especially powerful at feature selection, so we used it to find better subsets of input variables.

GA, a search algorithm based on concepts of natural selection and genetics, was officially introduced by Holland in the 1990s [27]. The underlying principles of GA are to generate an initial population of chromosomes (search solutions) and then use selection and recombination operators generate a new, more effective population which eventually will have the fittest chromosome (optimal value) among them.

The 10 steps of operation of ANN and GA hybrid intelligence are as follows.

**Step 1 (initialization of population).**

Generate an initial population of chromosomes which are bit strings of randomly generated binary values. The chromosome and population sizes that we used were 44 and 10, respectively.

**Step 2 (decoding).**

Decode chromosomes (bit strings) to find which input variables will be selected.

**Step 3 (ANN).**

Run three-layered feedforward ANN model to make prediction of next-day SET50 index. The parameters in the model that we used were the same as those reported by Inthachot et al. [12].

**Step 4 (fitness evaluation).**

Take the prediction accuracy of each chromosome from ANN as its fitness value for GA.

**Step 5 (stopping criterion).**

Determine whether to continue or exit the loop. The stopping criterion was not more than 10 generations.

**Step 6 (selection).**

Select chromosomes to cross over using tournament selection technique. A tournament selection involves running several tournaments on a few chromosomes chosen at random from the population. The winner of each tournament is selected for crossover.

**Step 7 (crossover).**

Apply an arithmetic crossover operator that defines a linear combination of two chromosomes.

**Step 8 (mutation).**

Inject new genes into the population with uniform mutation operator and generate a random slot number of the crossed-over chromosome as well as flip the binary value in that slot.

**Step 9 (replacement).**

Replace old chromosomes with two best offspring chromosomes for the next generation.

**Step 10 (loop).**

Go to Step 2.

**Figure 1:**
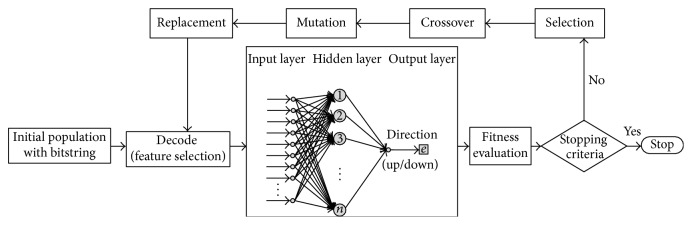
Steps of operation of ANN and GA hybrid intelligence.

All of the steps are shown in Figure 1.

### 3.3. Fitness Evaluation

We used accuracy to determine chromosome selection (subsets of input variables)—chromosomes that would generate the next generation—as well as to measure the performance of the prediction model. Fitness values in GA were taken as the accuracy values that can be calculated as below:

(1) $$\begin{array}{c} \text{Accuracy}=\frac{\text{TP}+\text{TN}}{\text{TP}+\text{TN}+\text{FP}+\text{FN}}, \end{array}$$

where TP is true positive, FP is false positive, TN is true negative, and FN is false negative.

## 4. Results and Discussion

A hybrid intelligence of ANN and GA models was developed for predicting SET50 index movement during 2009–2014. Each year's trading data during this period were converted into 11 technical indicators, each of which represented by 4 input variables based on different lengths of past time spans, and hence 44 input variables. All input variables were identically normalized and subsets of them were selected by GA and imported into ANN that would use them to make stock index movement prediction. Fivefold cross-validation runs were made to guarantee reliability. This hybrid intelligence was coded and run in a MATLAB software environment.

**Figure 2:**
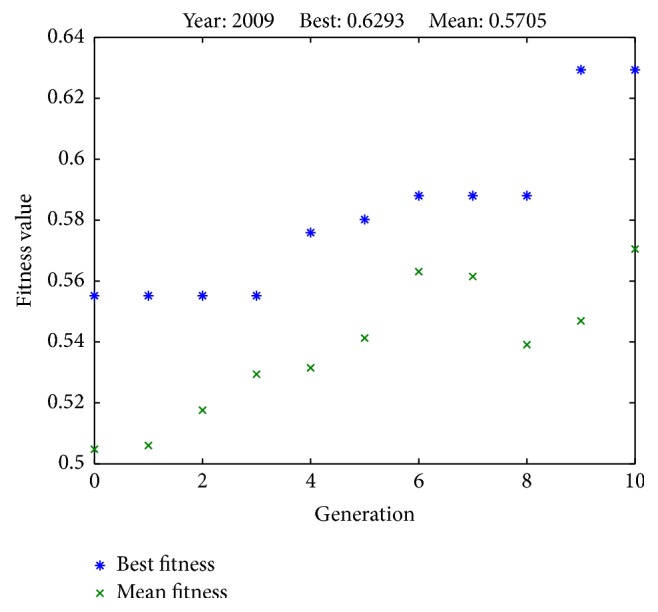
Fitness values in each generation (predicting SET50 index in 2009).

**Figure 3 fig3:**
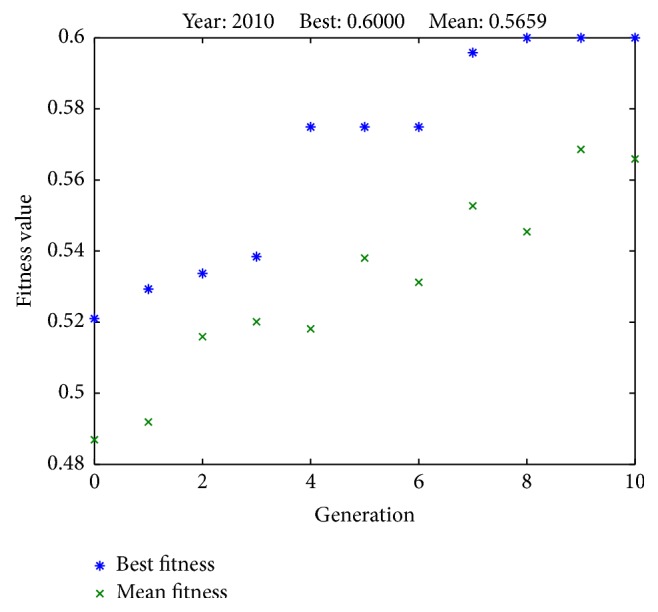
Fitness values in each generation (predicting SET50 index in 2010).

**Figure 4 fig4:**
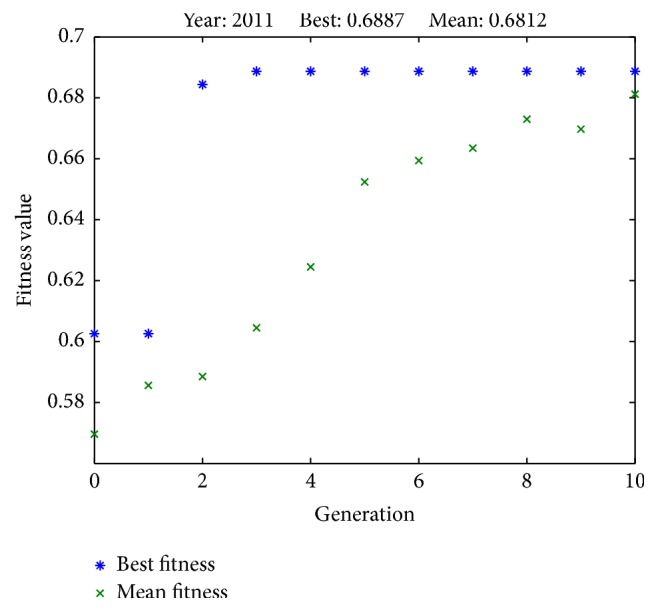
Fitness values in each generation (predicting SET50 index in 2011).

**Figure 5 fig5:**
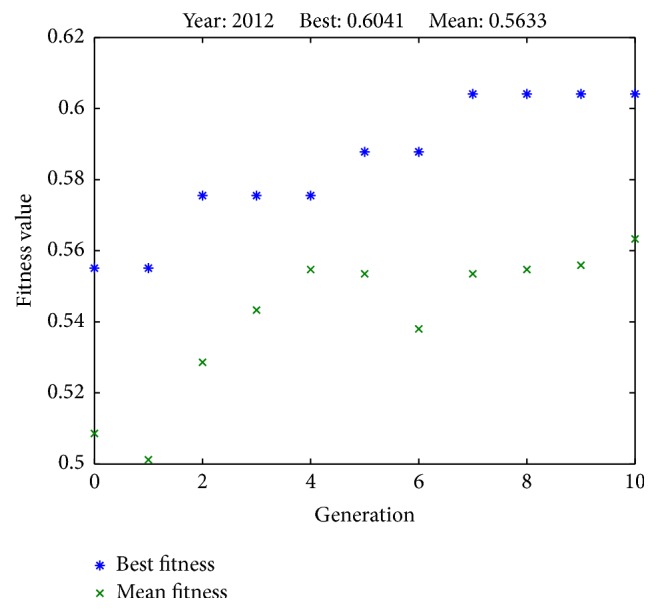
Fitness values in each generation (predicting SET50 index in 2012).

**Figure 6 fig6:**
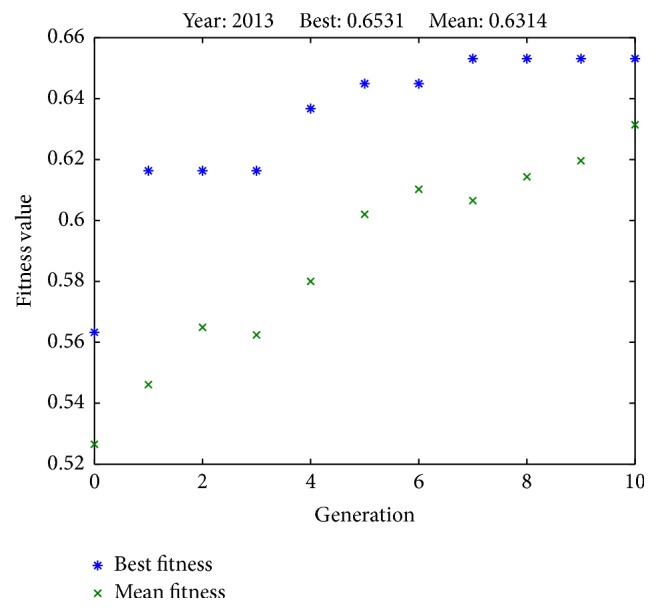
Fitness values in each generation (predicting SET50 index in 2013).

**Figure 7:**
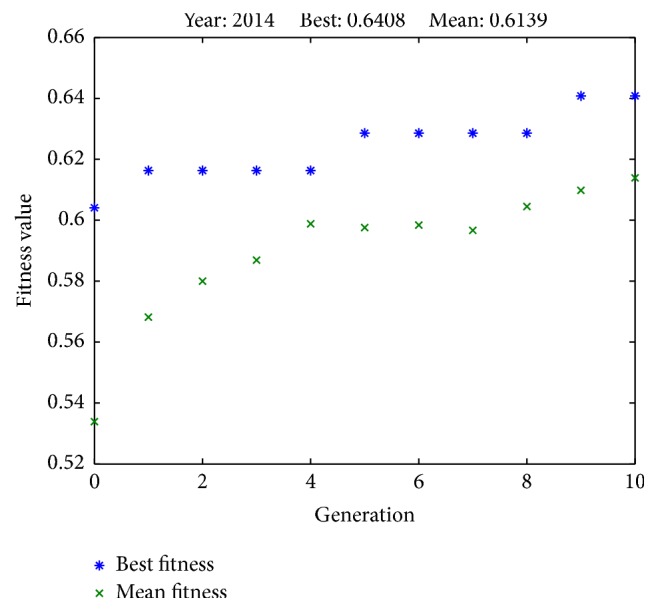
Fitness values in each generation (predicting SET50 index in 2014).

Results from successful runs are presented in Figures 2 [Fig fig3] [Fig fig4] [Fig fig5] [Fig fig6]–7 and Table 4. Figures 2 [Fig fig3] [Fig fig4] [Fig fig5] [Fig fig6]–7 illustrate the best fitness value (which reflects the prediction accuracy) achieved in each generation for each year of prediction.

The trend prediction accuracy performance of our proposed method was compared to another study [12] only because even though there have been several works on SET, all of them reported either the mean square error of stock price or stock price index [17, 28–30]. Moreover, it was not compared to those of any binary choices model because it has been widely reported in the literature that their prediction performances were inferior to ANN in regard to stock market prediction [2, 11, 12].

Table 4 compares the accuracies achieved by the model that Inthachot et al. [12] used in their study and those achieved by our hybrid intelligence of ANN and GA models. It can be seen that the model that Inthachot et al. [12] used achieved its lowest prediction accuracy of 52.57% for the year 2010, highest accuracy of 59.86% for the year 2011, and average accuracy of 56.58%. On the other hand, our hybrid intelligence achieved its lowest prediction accuracy of 60.00% for the year 2010, highest accuracy of 68.87% for the year 2011, and average accuracy of 63.60%.

This study's hybrid intelligence predicted more accurately than the model used by Inthachot et al. [12] for every year during the selected period with the lowest percentage improvement of 8.0293%, the highest of 15.0518%, and the average improvement of 12.4011%. In order to confirm this conclusion statistically, we compared them using a *t*-test at 0.05 level of significance and found that the *P* value of the right tail was 0.0009; hence the conclusion is valid.

## 5. Conclusion

In this study, we developed a hybrid intelligence of ANN and GA models for predicting SET50 stock index movement and tested it on a large set of past stock trading data. The purpose of the development was to achieve a better prediction accuracy than that obtained by a previous ANN model that we have developed [12]. Test results show that the hybrid intelligence has accomplished this purpose, gaining an average improvement of 12.4011%. It is 63.60% average prediction accuracy; however, it was still not very high and we are looking into combining ANN with other machine learning models in order to gain a higher prediction accuracy.

## Figures and Tables

**Table 1:** The number of up and down movements of SET50 index during 2009–2014.

Year	Up (times)	Up (%)	Down (times)	Down (%)	Total
2009	137	56.38	106	43.62	243
2010	138	57.02	104	42.98	242
2011	119	48.77	125	51.23	244
2012	140	57.14	105	42.86	245
2013	126	51.43	119	48.57	245
2014	135	55.10	110	44.90	245
Total	795	54.30	669	45.70	1,464

**Table 2:** The number of up and down movements of the whole set of daily index in 5 cross-validation runs.

Year	Five runs of cross-validation										Total
	1st run		2nd run		3rd run		4th run		5th run		
	Up	Down	Up	Down	Up	Down	Up	Down	Up	Down	
2009	27	22	31	18	24	25	32	16	23	25	243
2010	28	20	30	18	37	11	17	32	26	23	242
2011	26	23	25	24	27	22	22	27	19	29	244
2012	30	19	22	27	30	19	24	25	34	15	245
2013	28	21	27	22	23	26	24	25	24	25	245
2014	26	23	23	26	36	13	19	30	31	18	245
Total	165	128	158	135	177	116	138	155	157	135	1,464

**Table 3:** Technical indicators used in this study and their equations [2, 18].

Indicator name	Equation	Level (*n*)	Total
Simple *n*-day moving average	$\frac{C_{t}+C_{t-1}+\cdots +C_{t-n-1}}{n}$	3, 5, 10, 15	4
Weighted *n*-day moving average	$\frac{{(n)C}_{t}+{(n-1)C}_{t-1}+\cdots +C_{t-(n-1)}}{n+\left(n-1\right)+\cdots +1}$	3, 5, 10, 15	4
Momentum	*C*_*t*_ − *C*_*t*−*n*_	3, 5, 10, 15	4
Stochastic K%	$\frac{C_{t}-{LL}_{t-(n-1)}}{{HH}_{t-(n-1)}-{LL}_{t-(n-1)}}\times 100$	3, 5, 10, 15	4
Stochastic D%	$\frac{\sum_{i=0}^{n-1}K_{t-i}\%}{n}$	3, 5, 10, 15	4
Relative Strength Index (RSI)	$100-\frac{100}{1+(\sum_{i=0}^{n-1}{(\text{UP}}_{t-i}/n))/(\sum_{i=0}^{n-1}\left({\text{DW}}_{t-i}/n\right))}$	3, 5, 10, 15	4
Moving Average Convergence Divergence (MACD)	${\text{MACD}\left(n\right)}_{t-1}+\frac{2}{n+1}\times \left({\text{DIFF}}_{t}-{\text{MACD}\left(n\right)}_{t-1}\right)$	3, 5, 10, 15	4
Larry William's R%	$\frac{H_{n}-C_{t}}{H_{n}-L_{n}}\times -100$	3, 5, 10, 15	4
Commodity Channel Index (CCI)	$\frac{M_{t}-{SM}_{t}}{0.015D_{t}}$	3, 5, 10, 15	4
Rate of change	$\frac{C_{t}-C_{t-n}}{C_{t-n}}\times 100$	3, 5, 10, 15	4
Average Directional Index (ADX)	$\text{SMA}\left(\frac{{+\text{DI}}_{n}-({-\text{DI}}_{n})}{+{\text{DI}}_{n}+(-{\text{DI}}_{n})}\right)$	3, 5, 10, 15	4
Total			44

Note: *n* is *n*-day period times ago; *C*_*t*_ is closing price; *L*_*t*_ is low price at time *t*; *H*_*t*_ is high price at time *t*; DIFF = EMA(12)_*t*_ − EMA(26)_*t*_; EMA is exponential moving average; EMA(*k*)_*t*_ = EMA(*k*)_*t*−1_ + ∝(*C*_*t*_ − EMA(*k*)_*t*−1_); ∝ is smoothing factor = 2/(1 + *k*); *k* = 10 in *k* −day exponential moving average; *LL*_*t*_ and *HH*_*t*_ are the lowest low and highest high in the last *t* days, respectively; *M*_*t*_ = (*H*_*t*_ + *L*_*t*_ + *C*_*t*_)/3; *SM*_*t*_ = ∑_*t*=1_^*n*^ *M*_*t*−*i*+1_/*n*; *D*_*t*_ = ∑_*i*=1_^*n*^|*M*_*t*−*i*+1_ − *SM*_*t*_|/*n*; UP_*t*_ is upward index change at time *t*, DW_*t*_ is downward index change at time *t*; +DI_*n*_ is plus directional indicator and −DI_*n*_ is minus directional indicator.

**Table 4:** Prediction performances of Inthachot et al. [12] model and this study's model.

Year	Accuracy		
	Inthachot et al. [12]	This study	Percentage increase
2009	0.5602	0.6293	12.3349%
2010	0.5257	0.6000	14.1335%
2011	0.5986	0.6887	15.0518%
2012	0.5592	0.6041	8.0293%
2013	0.5714	0.6531	14.2982%
2014	0.5796	0.6408	10.5590%
Average	0.5658	0.6360	12.4011%
